# Severe Hyperthyroidism With Transient Hepatocellular Injury and Gallstone-Related Pancreatitis in Graves’ Disease: A Case Report

**DOI:** 10.7759/cureus.95835

**Published:** 2025-10-31

**Authors:** Sathia Narayanan Mannath, Muhammed Rinas, Rekha Kethohalli Shivamurthy, Ebtihal Elfadoul, Cornelius Fernandez James

**Affiliations:** 1 Department of Endocrinology and Metabolism, Pilgrim Hospital, United Lincolnshire Teaching Hospitals NHS Trust, Boston, GBR; 2 Department of General Internal Medicine, Pilgrim Hospital, United Lincolnshire Teaching Hospitals NHS Trust, Boston, GBR

**Keywords:** acute intrahepatic cholestasis, gallstone pancreatitis, grave's, hepatic enzyme derangement, hyperthyroidism

## Abstract

Graves’ disease is an autoimmune thyroid disorder that can present with multisystem involvement, including transient hepatocellular dysfunction. While mild liver enzyme elevation is common in thyrotoxicosis, severe hepatic impairment and associated complications remain rare. We report the case of a 29-year-old man with newly diagnosed Graves’ disease who initially presented with marked weight loss, palpitations, and biochemical thyrotoxicosis. He subsequently developed acute right upper quadrant pain, with imaging features suggestive of gallstone-related acute pancreatitis and deranged liver function tests. The hepatic impairment was attributed to possible gallstone migration in the context of rapid weight loss from hyperthyroidism, rather than thionamide-induced hepatotoxicity. This case highlights the complex interplay between hyperthyroidism and transient hepatocellular injury. It emphasizes the importance of considering thyroid dysfunction in the differential diagnosis of unexplained hepatic abnormalities, as well as the need for careful monitoring when initiating antithyroid therapy in patients with baseline liver dysfunction. Furthermore, it demonstrates that rapid metabolic shifts in untreated hyperthyroidism may predispose patients to gallstone formation and subsequent pancreatitis.

## Introduction

Graves’ disease is an autoimmune condition in which the body's immune system produces thyroid-stimulating immunoglobulins that bind to and activate the thyroid-stimulating hormone (TSH) receptor on thyroid follicular cells. This inappropriate stimulation results in excessive synthesis and release of thyroid hormones, leading to a state of hyperthyroidism. Although Graves’ disease primarily targets the thyroid gland, its effects are not confined to the thyroid gland alone. The metabolic consequences of hyperthyroidism can influence multiple organ systems, including the liver [1]. Hepatic involvement in Graves’ disease can range from mild elevations in liver enzymes to more severe manifestations such as cholestasis or hepatic failure. In some cases, liver dysfunction may precede the overt signs of hyperthyroidism, making it a potential early indicator of underlying thyroid pathology. Several mechanisms may contribute to hepatic impairment [2].

Liver dysfunction is a clinically significant impairment of hepatic function, which affects essential processes such as metabolism, detoxification, protein synthesis, and bile production. It is typically supported by biochemical, clinical, or histological findings, including markedly abnormal liver function tests (LFTs), jaundice, coagulopathy, hypoalbuminemia, or hepatic encephalopathy. In contrast, mild or transient elevations in transaminases (ALT and AST) often reflect hepatocellular injury without true dysfunction. These elevations may arise from a range of benign or reversible factors, such as medication use, alcohol consumption, intense physical activity, fatty liver disease, or systemic illness [3].

The case underscores the importance of assessing liver function in patients with hyperthyroidism before commencement and while on antithyroid treatment. A thorough understanding of the interplay between thyroid hormone excess and liver pathology is vital to ensure prompt, accurate diagnosis and effective multidisciplinary treatment.

## Case presentation

**Figure 1:**
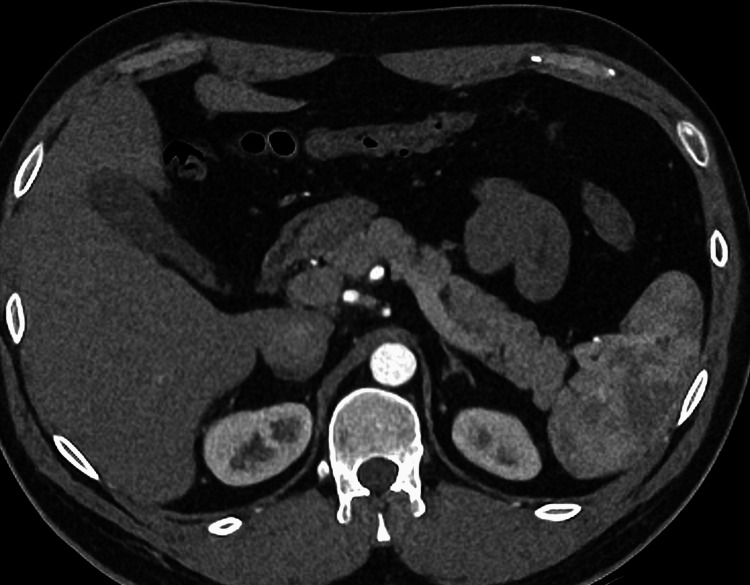
CT aortogram showing normal pancreas and gallbladder

A 29-year-old man presented to a nearby urgent treatment center with recurrent episodes of chest pain radiating to the back and shoulders, palpitations, shortness of breath, and elevated blood pressure. An ECG showed sinus tachycardia. The troponin levels were within normal limits, but mildly raised alanine aminotransferase (ALT), alkaline phosphatase (ALP), and gamma-glutamyl transferase (GGT) were noted. The patient was referred to the Accident & Emergency (A&E) department, where a CT aortogram (CTA) was performed and was found to be normal. On CTA, the gallbladder and pancreas were reported to be normal (Figure 1). It incidentally revealed two enhancing lesions in the liver on the arterial phase, suspecting hemangiomas. Though thyroid function tests were requested, the patient was discharged before the reports were processed. This had never been reviewed until the next presentation, many months later. There was a plan for an outpatient liver ultrasound (USG) in view of the possible hepatic hemangiomas.

**Figure 2:**
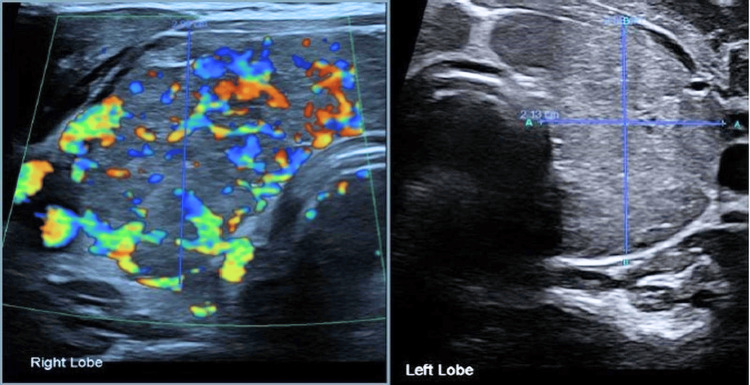
Thyroid ultrasound revealed diffusely enlarged and heterogeneous thyroid with markedly increased vascularity

Five months later, he presented to the A&E with symptoms suggestive of hyperthyroidism, including excessive sweating, hot flushes, poor sleep, tremor, palpitation, chest discomfort, dyspnea, and significant unintentional weight loss of approximately four stones (25.4 kgs) over four months. On examination, he was tachycardic, with fine hand tremors. His ECG showed sinus tachycardia. Thyroid function tests revealed a suppressed TSH with an elevated free T4 above 100 pmol/L, consistent with primary hyperthyroidism. Thyroid USG demonstrated a diffusely enlarged thyroid gland (Figure 2). 

Upon endocrine team review, the Burch-Wartofsky Point Scale (BWPS) score was 15, making a thyroid storm unlikely. The patient also reported retro-orbital discomfort. Ocular examination showed signs consistent with mild thyroid eye disease. Further work-up revealed elevated thyrotropin receptor antibody (TRAb) and thyroid peroxidase antibody, confirming the diagnosis of Graves’ disease with associated mild thyroid eye disease. 

Liver USG that was requested from a previous hospital visit happened along with thyroid USG, and it revealed the presence of multiple gallstones with a normal common bile duct (CBD) without any evidence of acute calculous cholecystitis. The patient was free of gallstone-related symptoms. As his LFTs were normal, medical therapy with carbimazole 40 mg once daily and propranolol 10 mg three times daily was initiated, and he was discharged.

**Figure 3:**
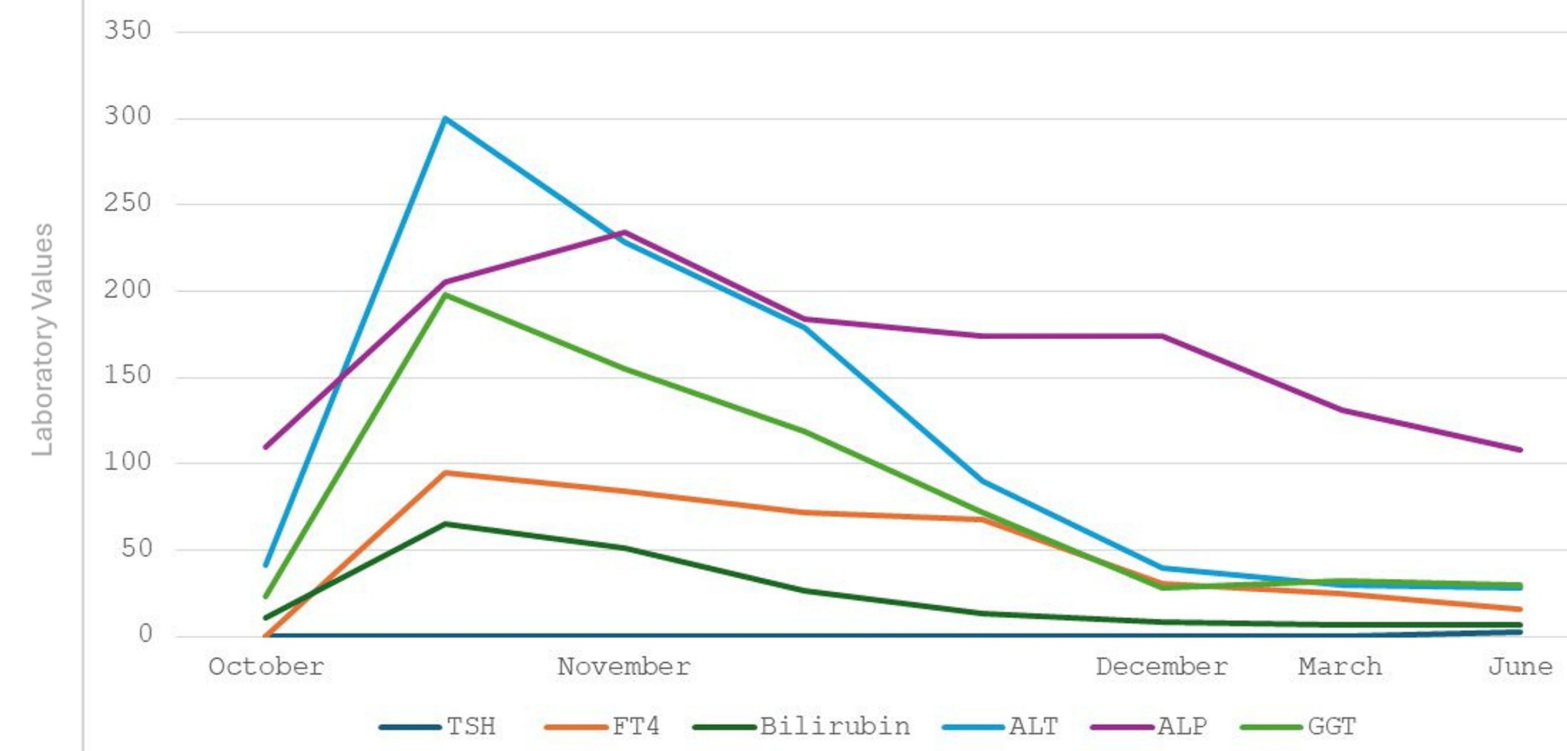
Trends in TSH, free T4, bilirubin, ALT, ALP, and GGT since the initiation of carbimazole TSH: Thyroid-Stimulating Hormone, ALT: Alanine Aminotransferase, ALP: Alkaline Phosphatase, GGT: Gamma-Glutamyl Transferase

Approximately three weeks later, the patient was admitted under the surgical team with an acute onset of severe right upper quadrant and epigastric pain radiating to the back and right shoulder, accompanied by multiple episodes of vomiting, dark urine and pale stools. On clinical examination, there was tenderness over the right hypochondrium and epigastrium, with mild Icterus. Laboratory investigations revealed raised amylase, together with newly raised bilirubin, ALT, ALP, and GGT levels (Table 1, Figure 3). Bilirubin fractionation is not routinely performed in our Trust, especially in surgical wards, unless clearly indicated.

**Table 1:** Trend in laboratory markers since the initial presentation in May TSH: Thyroid-Stimulating Hormone, FT4: Free T4, TRAb: Thyrotropin Receptor Antibodies, TPO Ab: Thyroid Peroxidase Antibodies, ALT: Alanine Aminotransferase, ALP: Alkaline Phosphatase, GGT: Gamma-Glutamyl transferase

	19/05	09/10	31/10	01/11	02/11	11/11	06/12	05/03	14/06
TSH (0.27-4.5 mU/L)	<0.01	<0.01	<0.01	<0.01	<0.01	<0.01	<0.01	<0.01	2.3
FT4 (11-24 pmol/l)	34.8	>100	95.2	84.1	71.4	67.8	30.3	24.4	16
TRAb (0-1.74 U/L)	12.6	34.8	-	-	-	-	-	-	-
TPO Ab (0-34 IU/L)	-	76	-	-	-	-	-	-	-
Bilirubin (0-21 umol/L)	5	11	65	51	26	13	8	7	7
ALT (0-41 U/L)	88	41	300	228	179	90	40	30	28
ALP (30-130 U/L)	94	110	205	234	184	174	174	131	108
GGT (10-71 U/L)	130	25	198	155	119	72	28	32	30
Amylase (28-100 U/L)	-	-	49	628	155	-	-	-	-

**Figure 4:**
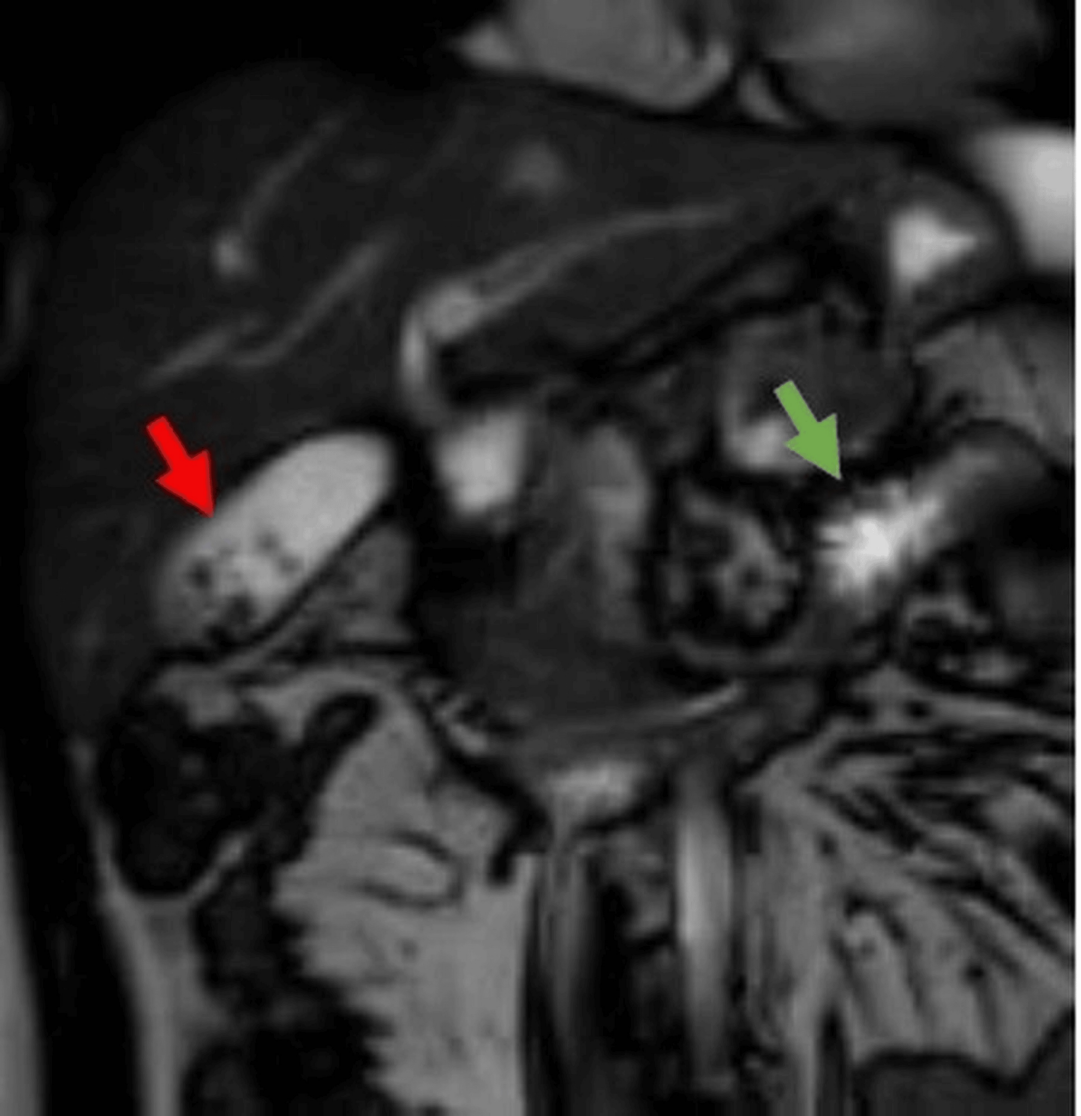
MRCP showing multiple gall bladder calculi (red arrow) with peri-pancreatic fat stranding (green arrow) and free fluid due to acute pancreatitis MRCP: Magnetic Resonance Cholangiopancreatography

Magnetic resonance cholangiopancreatography (Figure 4) confirmed the presence of multiple gallstones and peripancreatic inflammatory changes consistent with acute pancreatitis, without evidence of CBD dilatation. The patient was diagnosed with gallstone pancreatitis and conservatively managed with intravenous fluids, antiemetics, analgesics, bowel rest, followed by gradual reintroduction of oral feeding, a low-fat diet and a short course of prophylactic antibiotics.

The endocrine team assessed the safety of antithyroid medication and advised continuing carbimazole. The endocrine team noted that the LFTs are rapidly improving, and there is an alternative cause attributable to the transient hepatocellular injury, rather than carbimazole-induced hepatotoxicity.

Four weeks after discharge, the surgical team arranged MRI pancreas which showed resolution of pancreatitis, but with persistent gallstones. They planned for cholecystectomy once thyroid function is stabilized. Serial monitoring of thyroid and LFTs showed biochemical improvement. The patient underwent cholecystectomy four months later. The patient is currently under regular follow-up with the endocrinology team and is maintained on carbimazole 10 mg daily with ongoing blood monitoring. He is clinically well and reports no current symptoms.

## Discussion

Transient hepatocellular injury in patients with Graves’ disease and other causes of hyperthyroidism

Transient hepatocellular injury in patients with Graves’ disease and other causes of hyperthyroidism can arise through several interrelated mechanisms, encompassing both direct metabolic effects of excess thyroid hormone and indirect effects secondary to antithyroid drugs (ATDs), as well as from various systemic complications [2]. A thorough understanding of the interplay between thyroid hormone excess and liver pathology is vital to ensure prompt, accurate diagnosis and effective multidisciplinary treatment.

Direct hepatic effects of thyrotoxicosis

Excess thyroid hormones in hyperthyroidism significantly raise basal metabolic activity and hepatic oxygen demand. However, this is not matched by a corresponding increase in hepatic blood flow, resulting in centrilobular hypoxia and, in some cases, necrosis. This hypoxic injury may impair bile acid transport, leading to intrahepatic cholestasis. Histology of the liver may show hepatocellular fatty infiltration, nuclear irregularity, hyperchromasia, and cytoplasmic vacuoles [4].

Transient hepatocellular injury affects up to one-third of hyperthyroid patients and commonly manifests as elevated transaminases or cholestatic enzyme patterns, both of which typically improve with the restoration of euthyroidism [5]. Although routine liver function monitoring is not universally recommended, baseline liver function testing is advisable before initiating ATDs. Thionamides should be avoided if transaminases exceed five times the upper limit of normal and discontinued if levels rise more than three fold during treatment [5]. Baseline liver function testing is highly relevant in patients with pre-existing hepatic dysfunction. All patients on thionamides should be counseled to report symptoms of liver injury, including jaundice, fatigue, and dark urine [6].

Cardiac-related hepatic injury in thyrotoxic heart failure

Thyrotoxicosis impacts the cardiovascular system both centrally and peripherally. It increases heart rate and contractility via direct stimulation of the sinoatrial node and myocardium. Peripheral vasodilation lowers vascular resistance and diastolic pressure, activating the renin-angiotensin-aldosterone system, which promotes sodium and fluid retention, increasing preload and cardiac output [7]. This hyperdynamic state can raise cardiac output to 2-3 times normal, leading to high-output heart failure in approximately 6% of patients [8]. A smaller subset (1%) may develop a high-output thyrotoxic cardiomyopathy, or a low-output dilated cardiomyopathy, the latter often associated with chronic tachyarrhythmias or myocarditis [8]. Excessive cardiac output may also overload the right ventricle, causing pulmonary hypertension and, in rare cases, isolated right ventricular dysfunction. In hyperthyroid states, the resulting hyperdynamic circulation can progress to congestive heart failure, particularly in those with pre-existing cardiac disease. Right-sided heart failure may lead to hepatic congestion and liver dysfunction, presenting with features resembling viral or toxic hepatitis with marked transaminase elevation, jaundice, hepatomegaly, ascites, and coagulopathy due to impaired hepatic synthesis [9].

Hepatotoxicity induced by antithyroid medications

Both propylthiouracil (PTU) and carbimazole, or its active metabolite, methimazole, are associated with rare but potentially serious hepatic side effects. The incidence of clinically significant hepatotoxicity is estimated at 0.1-0.2% [10]. Risk factors include advanced age, higher ATD doses, elevated TRAb, and free T4 levels [10]. PTU is more commonly linked with hepatocellular necrosis. Its metabolism via hepatic glucuronidation may interfere with antioxidant enzymes, contributing to liver cell injury [11]. Hepatotoxicity from PTU typically develops after 2-3 months of treatment and tends to resolve within 2-5 months following drug cessation [12].

Carbimazole/methimazole is more often associated with cholestatic liver injury, without necrosis. It is believed that reactive metabolites, such as N-methylthiourea and glyoxal produced via cytochrome P450 enzymes, play a central role in this toxicity. Hepatic side effects from carbimazole usually occur within the first two weeks of therapy and may persist longer than those from PTU [12].

In severe cases, antithyroid drugs should be discontinued immediately, and agents such as cholestyramine may be used to manage cholestasis. Definitive therapy, such as radioiodine treatment or thyroidectomy, should then be considered [13]. Due to the potential to cause cross-reactivity between PTU and carbimazole, a switch between these agents in the event of drug-induced hepatotoxicity should be considered only if definitive therapies are either contraindicated or are not available.

Autoimmune liver diseases associated with thyrotoxicosis

Given the autoimmune basis of Graves’ disease, it may coexist with other autoimmune hepatobiliary disorders, including autoimmune hepatitis and primary biliary cholangitis. Up to 10% of patients with Graves’ disease may exhibit signs of such overlap, often presenting with elevated ALP levels and positive autoimmune markers including antinuclear antibody, smooth muscle antibody, and antimitochondrial antibody. In some cases, TRAb may directly bind to hepatic TSH receptors, contributing to immune-mediated hepatocyte injury [14].

Gallstone disease and biliary complications associated with thyrotoxicosis

Gallstone formation, though classically linked to hypothyroidism, may also occur in hyperthyroidism, especially in elderly women with risk factors such as obesity, rapid weight loss, and sedentary lifestyle [13]. Whereas the gallstones in hypothyroidism are due to enhanced cholesterol synthesis, those in hyperthyroidism are due to enhanced cholesterol metabolism from over-expression of hepatic nuclear receptor genes like liver X receptor alpha (LXRα) and retinoid X receptor gene (RXR) [15]. Additionally, hyperthyroidism increases bile acid secretion, and reduces gallbladder contractility in response to cholecystokinin, thereby promoting bile stasis and cholesterol supersaturation [13]. Moreover, decreased smooth muscle tone, including sphincter of Oddi relaxation, can facilitate stone migration into the biliary and pancreatic ducts, potentially leading to acute pancreatitis [16].

Thyroid storm and liver dysfunction

Although this patient did not meet the diagnostic criteria for thyroid storm, it is important to recognize that thyroid storm, an acute, life-threatening exacerbation of thyrotoxicosis characterized by multiorgan dysfunction and systemic decompensation, may be associated with hepatocellular injury [17]. It typically arises in individuals with untreated/undertreated hyperthyroidism, most commonly Graves’ disease, and is often precipitated by infection, surgery, trauma, or abrupt cessation of ATDs. Clinically, it presents with high fever, profuse sweating, marked tachycardia, arrhythmias such as atrial fibrillation, altered mental status, gastrointestinal symptoms (e.g., diarrhea, vomiting), and signs of cardiac or hepatic failure. The BWPS is a widely used tool to assess the probability of thyroid storm; a score ≥45 is highly suggestive. Liver dysfunction in thyroid storm can present as cholestasis, transaminitis, coagulopathy, or in rare cases, fulminant hepatic failure [3,17].

Factors unrelated to thyrotoxicosis: sepsis, systemic illness, alcohol and drugs

Systemic infections or sepsis can amplify pre-existing hepatic dysfunction in patients with uncontrolled hyperthyroidism. Additionally, alcohol or concurrent hepatotoxic medications may act as underlying hepatic stressors, lowering the threshold for liver injury during thyrotoxic states [18]. Metabolic dysfunction-associated steatotic liver disease can be observed in hyperthyroid patients with type 2 diabetes mellitus, obesity, and/or metabolic syndrome.

In the context of our case, a challenging decision for us was whether to use ATDs knowing that they may cause or exacerbate hepatocellular or cholestatic liver injury. Although imaging did not demonstrate CBD obstruction, the temporal association between the pancreato-biliary symptoms (right upper quadrant/epigastric pain, dark urine, and pale stools) and acute liver enzyme elevation associated with raised amylase levels on a background of gallstone disease strongly suggests gallstone pancreatitis from probable migration of gallbladder calculi as a cause of transient hepatocellular injury. This is supported by the fact that the LFTs improved following the resolution of pancreatitis despite continued use of ATDs.

A recent systematic review and meta-analysis reported normalization of ALT, AST, ALP, bilirubin, and GGT levels in 83%, 87%, 53%, 50%, and 70% of patients, respectively, following ATD treatment [2]. Although many clinicians monitor liver function during ATD therapy, evidence for its effectiveness in detecting early liver injury is limited. The ATA guidelines neither recommend for nor against routine monitoring but emphasize patient education on hepatotoxicity symptoms and prompt discontinuation of therapy if jaundice, malaise, dark urine, or pale stools occur [5].

The CTA done in May during the first presentation, during which hyperthyroidism was missed, has not shown any evidence of gallstones. CT (39-75%) is less sensitive compared to USG (95%) in detecting gallstones [19]. The absence of gallstones could indicate, most probably that the stones were not radio-opaque or less probably that they have formed secondary to hyperthyroidism in the subsequent five months, as hyperthyroidism may itself indirectly contribute to gallstone formation (Figure 1). The probability of hyperthyroidism as a cause of gallstones due to the short duration of hyperthyroidism. However, in our patient, it is more probable that the hyperthyroidism-mediated sphincter of Oddi relaxation might have facilitated the stone migration into the pancreatic duct, potentially leading to acute pancreatitis [15].

## Conclusions

The transient hepatocellular injury in the context of Graves’ disease could have a complex and multifactorial pathophysiology. Given the broad differentials, early recognition and careful evaluation of liver abnormalities in hyperthyroidism patients are crucial for guiding safe and effective management. Baseline and periodic liver function testing, especially in patients with preexisting hepatic risk factors, can help identify early signs of liver involvement or drug-induced injury. Clinicians should be alert to symptoms of hepatic decompensation and tailor treatment accordingly or opt for non-hepatotoxic alternatives including radioiodine or surgery. A multidisciplinary approach involving endocrinology, hepatology, cardiology, and, in some cases, general surgery, ensures comprehensive care. Such collaboration enables prompt intervention, minimizes the risk of long-term hepatic damage, and improves outcomes in patients with Graves’ disease presenting with transient hepatocellular injury. ATDs may still be used with caution if liver enzyme abnormalities are mild and stable with monitoring of liver function tests, especially if an alternative etiology is found. 
